# Forest Connectivity Regions of Canada Using Circuit Theory and Image Analysis

**DOI:** 10.1371/journal.pone.0169428

**Published:** 2017-02-01

**Authors:** David Pelletier, Marc-Élie Lapointe, Michael A. Wulder, Joanne C. White, Jeffrey A. Cardille

**Affiliations:** 1 Department of Natural Resource Sciences and McGill School of Environment, McGill University, Montreal, QC, Canada; 2 Département de Mathématiques, Collège Montmorency, Laval, QC, Canada; 3 Canadian Forest Service (Pacific Forestry Centre), Natural Resources Canada, Victoria, BC, Canada; University of Guelph, CANADA

## Abstract

Ecological processes are increasingly well understood over smaller areas, yet information regarding interconnections and the hierarchical nature of ecosystems remains less studied and understood. Information on connectivity over large areas with high resolution source information provides for both local detail and regional context. The emerging capacity to apply circuit theory to create maps of omnidirectional connectivity provides an opportunity for improved and quantitative depictions of forest connectivity, supporting the formation and testing of hypotheses about the density of animal movement, ecosystem structure, and related links to natural and anthropogenic forces. In this research, our goal was to delineate regions where connectivity regimes are similar across the boreal region of Canada using new quantitative analyses for characterizing connectivity over large areas (e.g., millions of hectares). Utilizing the Earth Observation for Sustainable Development of forests (EOSD) circa 2000 Landsat-derived land-cover map, we created and analyzed a national-scale map of omnidirectional forest connectivity at 25m resolution over 10000 tiles of 625 km^2^ each, spanning the forested regions of Canada. Using image recognition software to detect corridors, pinch points, and barriers to movements at multiple spatial scales in each tile, we developed a simple measure of the structural complexity of connectivity patterns in omnidirectional connectivity maps. We then mapped the Circuitscape resistance distance measure and used it in conjunction with the complexity data to study connectivity characteristics in each forested ecozone. Ecozone boundaries masked substantial systematic patterns in connectivity characteristics that are uncovered using a new classification of connectivity patterns that revealed six clear groups of forest connectivity patterns found in Canada. The resulting maps allow exploration of omnidirectional forest connectivity patterns at full resolution while permitting quantitative analyses of connectivity over broad areas, informing modeling, planning and monitoring efforts.

## Introduction

Forests provide a broad range of ecosystem goods and services [1], and as the Earth's human population increases, so do demands on the planet's forest resources [2]. Canada is a large forest nation, representing approximately 9% of the world's forests and 28% of the world's boreal forest [3, 4]. Canada's forests are dynamic with a wide variety of disturbances resulting from both natural and anthropogenic causes [5], and the connectivity of forested landscapes for vagile organisms is likely in constant flux, driven by both disturbance and regrowth [6, 7]. Meanwhile, climate change is also impacting Canada's forests; together, these factors indicate a need for understanding forest connectivity across broad spatial scales and demand increased monitoring efforts and adaptive management strategies [8].

Even under simplified assumptions of a static landscape, measurements of connectivity for ecological studies are still in development [9–12]. The ecosystem concept of ecological connectivity has been defined as the connectedness of ecological processes, such as energy flow through an interaction network wherein species are connected via trophic relationships [13]. Habitat connectivity is a species-specific concept defined as the potential for movements between habitat patches, and quantified at either patch or landscape scales [12, 14, 15]. Landscape connectivity is an anthropogenic construct, and refers broadly to the connectedness of vegetation cover on the landscape [16]. While landscape connectivity may equate to habitat connectivity for some species, increases or decreases in landscape connectivity are not necessarily beneficial nor detrimental for any specific species. Similarly, although increases in landscape connectivity are generally beneficial in the context of ecological connectivity and a positive correlation between landscape connectivity and ecological connectivity may often exist [16, 17], such as the facilitation of seed dispersal through corridors [18], the maintenance of some ecological processes may not be enabled solely by increases in landscape connectivity alone.

The application of circuit theory [19–21] in landscape ecology using the Circuitscape program [22, 23] has allowed researchers to more readily assess habitat connectivity and genetic connectivity in a wide variety of environments and time scales using a standard approach. First employed in ecology to better understand genetic differentiation among populations, circuit theory also been explored to study connectivity for animal movement. For instance, Koen et al. [24] used Circuitscape for development and validation of multispecies linkage maps and Breckheimer et al. [25] used it to evaluate the umbrella species concept for conservation and restoration. Many studies using Circuitscape focus largely on the resistance distance metric between selected habitat or dispersal patches and place less emphasis on the current density maps [26–29]. In these studies, there is often a strong correlation between genetic difference intra-species and the resistance distance between habitat patches.

Although resistance distance is a well-understood measure, the current density patterns produced by Circuitscape are a largely untapped resource for ecology. Recent research [30, 31] has allowed for the creation of omnidirectional connectivity maps that are structurally complex and illustrate many facets of movement beyond the resistance distance computed automatically by Circuitscape [22]. The connectivity surfaces reveal the structure of movement across the landscape, showing many features including pinch points, restricted corridors, and obstacles to movement at a range of scales. Omnidirectional maps of connectivity suggest multiple possible pathways through a landscape, which allows users to identify movement paths that would be difficult to envision if limited to observation, or rule-based interpretations, of land-cover maps. These features vary widely in quantity and location because of the wide variety of possible landscape compositions.

Identification of landscape features at a variety of scales is considered an important avenue of research in landscape ecology [32]. In circuit-based maps, which were developed much more recently than vector-based representations, one key goal is the identification of features that can be perceived by eye and have been shown to be relevant to movement [33]. In addition to addressing the difficult challenge of detecting barriers to flow in maps of connectivity [34], there has been some progress identifying pinch points in Circuitscape outputs [35]. The automatic detection of pinch points across large areas, however, has to date been primarily limited to identifying pixels with high current value across multiple runs of Circuitscape. Pinch points, as will be demonstrated in this research, can be detected computationally using the established image-processing algorithm SURF [36], which identifies visually distinctive features at a variety of spatial scales.

Large territories such as Canada comprise landscapes that reflect different climatic regions, geological formations, and vegetation configurations [37] that serve to influence animal movement. While many different landscape structural arrangements might result in the same resistance distance, each of these arrangements produces a distinct connectivity surface that reflects its particular characteristics of landscape composition and configuration. Over large and varied landscapes, it is possible to identify areas with similar connectivity patterns based on detected features and connectivity metrics, and then create groups of forest connectivity regions that have similar patterns. For species with a known home range, such information could be used for preservation and conservation, while in the case of migratory species, this information would be useful to preserve and / or create migratory corridors.

In this research, we demonstrate the generation of these information products. We develop and interpret an omnidirectional forest connectivity map of over 600 million hectares of forested ecosystems in Canada. Using the techniques outlined in Pelletier et al. [31], we describe the division of the landscape into tens of thousands of interlocking tiles, the computation and analysis of omnidirectional connectivity surfaces, and the characteristics of the national-scale mosaic. We then describe new techniques for assessing the features of these landscape connectivity surfaces representing ease of movement and the configuration of the current density surface, using the new measures to classify the entirety of forested Canada into distinct connectivity groups.

## Methods

The foundational data used for this study was the circa-2000 Earth Observation for Sustainable Development of Forests land-cover product (EOSD LC 2000) [38]. The EOSD LC 2000 was derived primarily from Landsat Enhanced Thematic Mapper (ETM+) imagery and was produced to characterize the entirety of Canada's forested ecosystems, representing about 60% of Canada's landmass. The 23-class land-cover map, which comprises most common land covers of Canada such as plains, wetlands, tundra and bare rock, includes 9 forest classes (coniferous, broadleaf, mixedwood with dense, open, and sparse canopy closure) and was developed with a 25m spatial resolution. Among many applications, the EOSD has been used to assess the fragmentation of Canada's forests [39], to identify representative forested landscapes of Canada [40], and to characterize Canada's forest fragmentation regimes [41].

Prior to running Circuitscape, the 23 EOSD land-cover classes were generalized to three classes: forest, non-forest, and "no data" as per Wulder et al. [39]. To create a resistance map for Circuitscape, each pixel in the EOSD dataset was assigned a resistance value to create a contrast between forested and non-forested areas, analogous to what would be perceived by a forest dwelling organism. Forest pixels were assigned a resistance of 1, while non-forest and no-data pixels were assigned a resistance of 500. This binary forest/nonforest map was consistent with the approach for applying Morphological Spatial Pattern Analysis in land-cover maps [42–44]. This considerable simplification enabled us to assess whether there was a first-order relationship between the composition and configuration of forested land cover and the many complex features detected in the resulting current density maps, as described below.

We computed a nationwide forest connectivity map using the tiling approach presented in Pelletier et al. [31]. After assigning resistance values to each pixel of the EOSD, we partitioned the nationwide map into square tiles of 1000 x1000 pixels (25km x 25km). Tiles made entirely of the "no data" class were excluded from further analysis. To avoid edge effects in the final combined outputs, a buffer of 1000 pixels was added around each tile before processing in Circuitscape, creating a total of roughly 15,000 tiles of 3000x3000 pixels that contained land-cover data. Where neighboring tiles were unavailable for assembly, the area without data was filled with resistance values for non-forest (500). All tiles were processed twice in pairwise mode in Circuitscape 3.5.4, in east-west (horizontal) and north-south (vertical) directions. After processing in Circuitscape, the buffer around each tile was removed and the tiles were reassembled into complete, seamless, north-south and east-west directional mosaics. The directional current density values were multiplied together to create an omnidirectional connectivity mosaic. We then removed the extra buffer tiles around the mosaic to match the original spatial extent of the EOSD LC 2000 dataset. This resulted in 9923 tiles that each showed omnidirectional connectivity at high resolution across Canada's forested ecosystems. For each execution of Circuitscape that formed the omnidirectional connectivity mosaic, Circuitscape generated a resistance distance value for movement across the tile. For each tile, we averaged the north-south and east-west resistance distance values together to create an omnidirectional resistance distance value, which corresponds conceptually to the overall ease of movement across each tile.

The resistance distance metric is related to the forest composition of each tile, but does not capture characteristics of the spatial patterns of flow on each tile’s current density map. We explored metrics that could represent the configuration of flow at multiple scales in a tile, in particular searching for a method to automatically identify pinch points across spatial scales. To identify features on each of the 9923 tiles, we used the Speeded Up Robust Features (SURF) [36] image recognition software. SURF was configured to recognize features having substantial contrast between low current density areas and medium and high current density areas of all sizes and shapes. For each tile, SURF indicated the size, orientation, and location of each of hundreds or thousands of features of potential interest in understanding landscape connectivity. Inspecting SURF’s results on hundreds of tiles, we explored several potential ways to straightforwardly summarize the structural complexity of connectivity patterns using the great wealth of information returned by SURF, considering the total number of features detected, the number of features in bins of different sizes, the size of the largest feature, and the number of features larger than a certain threshold. To represent the arrangement and complexity of such structural features, we chose the number of SURF features detected, since this metric was the simplest of the potential measures, was correlated with other potential measures, and captured the importance of both small and large features on an omnidirectional connectivity surface.

In order to evaluate our application of the Circuitscape metrics we developed, we then analyzed the distribution of connectivity data inside the 12 Canadian ecozones that comprised nearly all of the EOSD [37]. Ecozones represent the highest level in a hierarchical zonation framework for Canada and are distinguished by a range of biotic and abiotic factors. The twelve ecozones considered herein represent a broad range of forest types and conditions, from the coastal forests of the Pacific Maritime ecozone, to the northern boreal forests of the Taiga and Boreal Shield ecozones, to the mixedwood forests of Atlantic Canada. We compared ecozone-specific connectivity patterns with boxplots of resistance distance and structural complexity for landscapes in each ecozone. We then explored the patterns of connectivity using the affinity propagation algorithm [45] to identify two representative landscapes, with respect to forest connectivity, in each ecozone.

With resistance distance and the number of SURF features calculated for each tile in the national mosaic, we used the two measures to reveal regions where connectivity patterns are similar across the boreal region of Canada. First, Canada-wide maps were generated that show, for the first time, the tile-based resistance distance and structural complexity from the values returned by Circuitscape and SURF at the national scale. We used the *mclust* package in R 3.0.2 [46, 47] on the normalized values to look for an optimal number of clusters within the data; we chose six as a number of groups that could illustrate the variability in the set while maintaining a coherent spatial pattern. Again using the affinity propagation algorithm, we clustered the tiles into six connectivity groups based on tile resistance distance and structural complexity, independent of ecozone boundaries. These new forest connectivity regions each are represented by a single “Exemplar” landscape, provided as part of the affinity propagation algorithm’s execution. To help characterize the patterns revealed by these regions, we identified the five landscapes that had resistance distance and complexity that were the most similar to each Exemplar. Using these landscapes and the statistical characteristics of the clusters, we developed descriptions of each of the forest connectivity regions of forested Canada.

## Results

### National-scale omnidirectional connectivity mosaic

**Fig 1:**
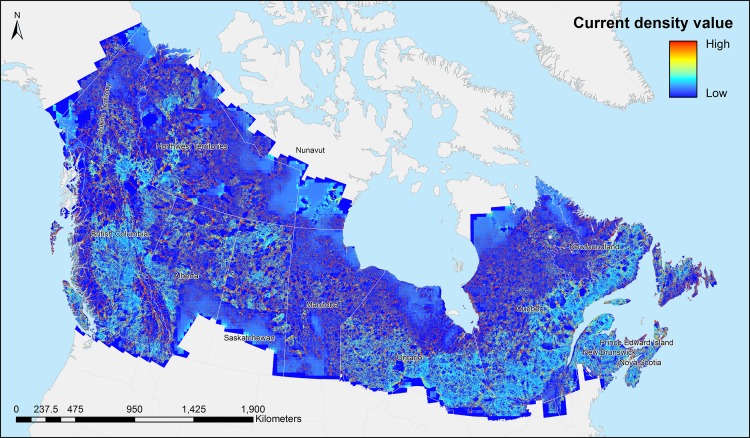
Omnidirectional connectivity mosaic of forested Canada at a resolution of 25 meters per pixel.

**Fig 2:**
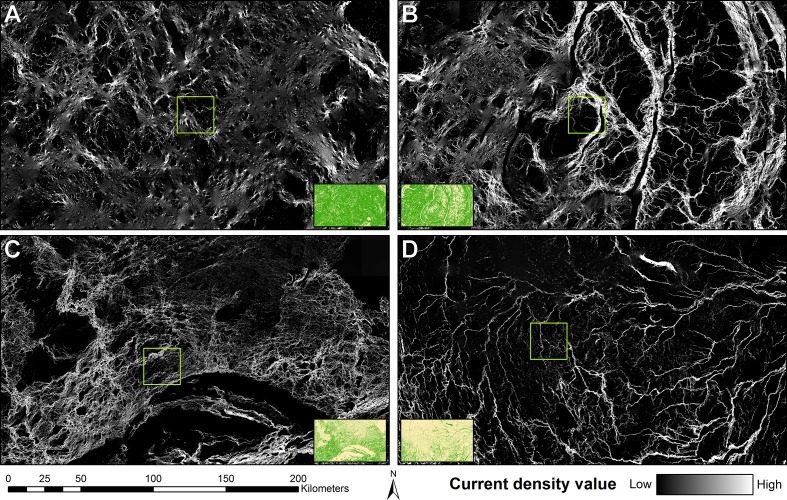
Sample areas from the Canada-wide current density mosaic. (a) Exemplar 2 of the Boreal Shield East ecozone (square green outline) and surrounding area. (b) Exemplar 2 of the Montane Cordillera ecozone and surrounding area. (c) Exemplar 2 from the Taiga Shield West ecozone and surrounding area. (d) Exemplar 2 of the Hudson Plains ecozone and surrounding area. Insets show the resistance maps for each of the areas surrounding and including the exemplars: forest in green and nonforest in yellow.

The omnidirectional connectivity mosaic (Fig 1) shows a variety of connectivity patterns across the full extent of boreal Canada. At the national scale, the connectivity mosaic is a mix of barriers (in black), relatively unconstrained flow (in gray), and pinch points [21, 31] (in white). Many densely forested areas, such as the southeastern part of Ontario and southern Québec (Fig 2A) show multiple paths and few constrained pinch points. The current density is spread around in multiple large paths and aggregates only around large obstacles such as the large lake surrounded by large areas of open non-forested landscape in the upper right part of the figure. While the area is not devoid of pinch points, they are small, spread around a greater landscape and are hourglass-shaped areas, indicating the connecting of two larger areas. Some narrow corridors channeling current are visible near large obstacles.

Connectivity patterns from the Montane Cordillera (Rocky Mountains, Fig 2B) are similar to other montane areas of western Canada. This area is comprised of multiple, interconnected and forested river valleys, visible as the bright paths in the middle and rightmost parts of Fig 2B. These areas are made of large parallel and forested corridors separated in the middle by a small or large river. Large parts of the landscape in this area are made of exposed rocks and, steep slopes and glaciers, which are visible as black areas bordering the forested valleys on the current density maps. This area is full of important movement corridors where current is forced to flow through and aggregate into high current density paths. A large number of pinch points are visible connecting these corridors together.

The Taiga Shield (Fig 2C) is diverse and has sparse forest cover. Large parts of this landscape are non-forested marshes and shrub land and it is covered by thousands of lakes of various sizes. The large black expanse of unconnected area visible in Fig 2C has no forest and shows as completely disconnected in the current density map. The upper middle part of the figure is made of numerous very small forest patches that are visible as small white speckles. Together these small forest patches are acting as stepping-stones and channel a lot of current flow. While most of the matrix is non-forested and provides high resistance to current flow in the model, current is forced to flow through it, and gives it a dark gray color. The southern and western parts of Fig 2C that are not covered by water are heavily forested and show up as corridors of various sizes and importance on the current density map. These corridors are interconnected by pinch points of varying sizes and channel high current flow.

The Hudson Plains is one of the largest contiguous wetland ecosystems on Earth and contains a large number of water bodies of various sizes and shapes (Fig 2D). Forest is spread around in thin bands bordering various rivers and meanders, visible as narrow white movement corridors in current density maps. These small corridors carry a lot of current flow and are interconnected by a number of pinch points. Some areas, such as the one in the center top of Fig 2D have very few sparse and small patches of forest that act as stepping stones for the current to flow through the landscape, and as in the Taiga Shield, the current flows between patches through non-conductive open areas, which makes it visible as dark-gray colored.

### Tile processing and analyses

**Fig 3:**
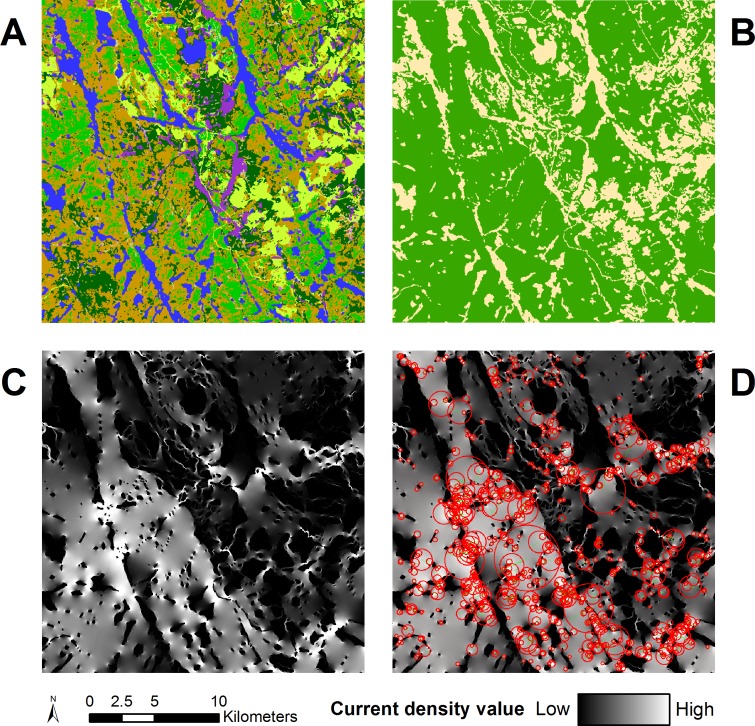
Processing and feature detection of a tile in the omnidirectional connectivity mosaic. (a) the original EOSD data for Exemplar 2 of the Boreal Shield East ecozone. (b) the resistance map where green is forest and tan is non-forest. (c) the omnidirectional current density map; (d) features detected by SURF, highlighted with red circles and sized to the feature’s size as identified by SURF.

Fig 3 shows each step of the tiling method and automatic feature detection with SURF. First is the EOSD land-cover classification (Fig 3A). In this landscape of the Boreal Shield ecozone, large number of lakes and rivers are visible in blue, as well as open areas in yellow and tan. A number of large marshes, visible in purple, are connected to the water areas. Forested areas are shown in shades of green and orange. This data was used for the reclassification into the resistance map shown in Fig 3B. The resistance map shows forested areas in green and all other areas as tan. The results from Circuitscape are shown in Fig 3C, the omnidirectional connectivity map was produced by combining the north-south and east-west Circuitscape runs. In the western part of the tile, large movement corridors are visible. The eastern part of the tile is more fragmented and result in a web of smaller, lower current density corridors. Several pinch points are visible in white. SURF (Fig 3D) can detect features (in red circles) at a variety of scales, which in omnidirectional connectivity maps range from bright pinch points a few pixels across, to large funnels spanning several kilometers.

**Fig 4:**
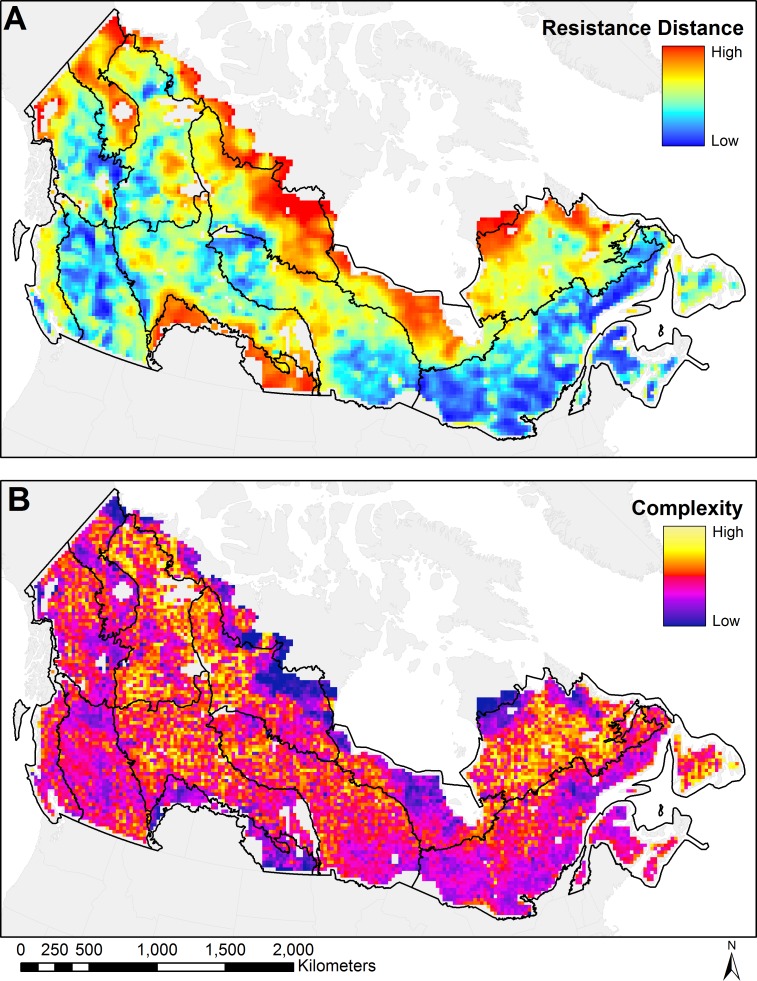
Measures of connectivity across Canada. (a) map of omnidirectional resistance distance for all tiles with non-zero forest cover; (b) complexity map of Canada for all tiles with non-zero forest cover. Black outlines show the Canadian ecozone boundaries.

Tile-level analysis highlights details that are not visible on the full mosaic of connectivity. Fig 4A shows connectivity in terms of omnidirectional resistance distance as computed by Circuitscape. Blue represents a low resistance distance, green and yellow represent a landscape that is more difficult for a forest dwelling organism to traverse, and red areas have little to no paths of current flow available. At the national scale, the heavily forested areas of Canada show low resistance distance, while non-forested regions such as the prairies and tundra show high resistance distance. However, areas with limited forest cover can still have easy traversal for a forest dwelling organism, such as is visible in the mountainous areas of western Canada, where long and wide forested river valleys interconnect large areas.

A tile-level map (Fig 4B) shows the number of features detected by the SURF image recognition software. Both tiles that are well connected and that have few corridors show up as various shades of purple in this figure, as they are both made of a generally homogeneous matrix that has few distinguishable features. Tiles with the most structural complexity in their current flow are those that have a mix of forest and non-forest covers and that have a high number of small obstacles, for example in Exemplars from the Boreal and Taiga Plains.

### Canadian ecozones analysis

**Fig 5:**
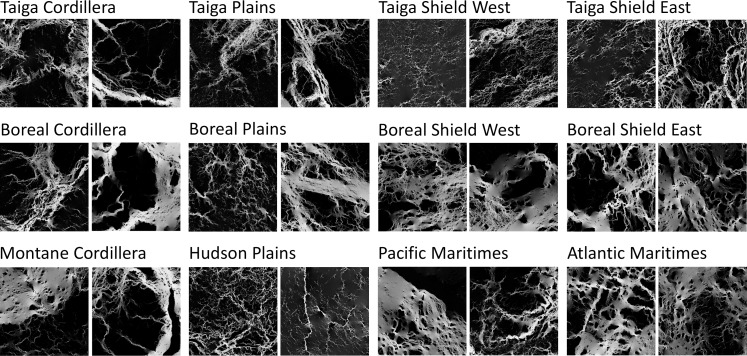
Forest connectivity exemplars for 12 Canadian ecozones, identified by resistance distance and number of identified features.

The Exemplars generated using the affinity propagation algorithm show typical forest connectivity patterns in each ecozone (Fig 5). Areas where there are few forest patches such as Exemplar 1 from the Taiga Shield West and Exemplar 2 from the Hudson Plains have low number of features. Areas that are partly covered by forest, such as Exemplars from the boreal and mountainous ecozones, show interesting configurations of features along large corridors. A wide variety of tiles from the Taiga ecozones and the Hudson Plains show multiple combinations of paths and obstacles and detected features tend to cluster around these high current density paths and pinch points. The tiles with the most detected features have forest cover of around 50% on their resistance maps and show a web of multiple different corridors such as Exemplar 2 from the Taiga Shield East and Exemplar 2 from the Boreal Plains.

**Fig 6:**
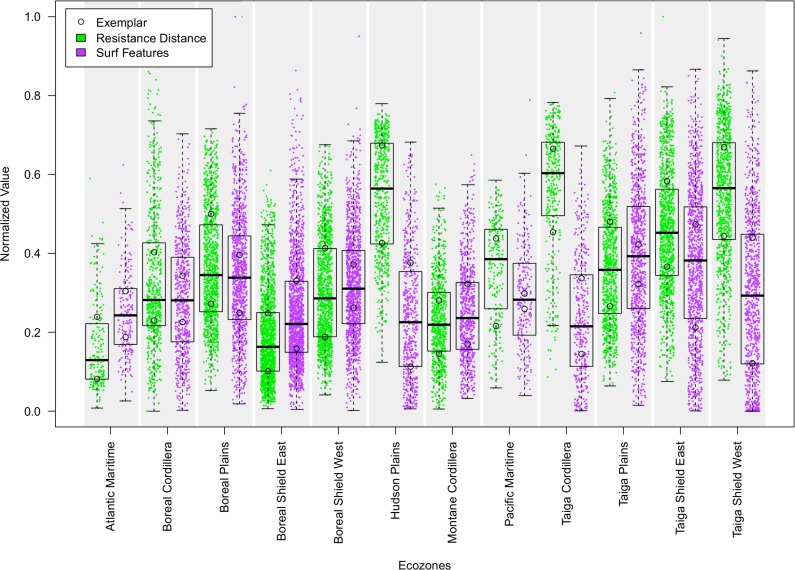
Resistance distance and SURF feature count for tiles in omnidirectional connectivity mosaic (625 km^2^ each).

Although ecozones are formed with the goal of having fairly uniform biotic and abiotic characteristics, each contains landscapes spanning nearly the full range of resistance distance values and complexity values found across Canada (Fig 4, Fig 6). Although differences among some resistance distance and complexity characteristics match ecozone boundaries (Fig 4), there is considerable variability in each zone. There are clear differences in resistance distances when crossing some ecozone borders, such as between the Hudson Plains and the eastern Boreal Shield. Similarly, there are some borders between ecozones that coincide with clear changes in the structural complexity of flow patterns, such as between the Boreal Plains and the Montane Cordillera. Overall, however, ecozone borders do not clearly delineate these connectivity characteristics.

### Canadian forest connectivity regions

**Fig 7:**
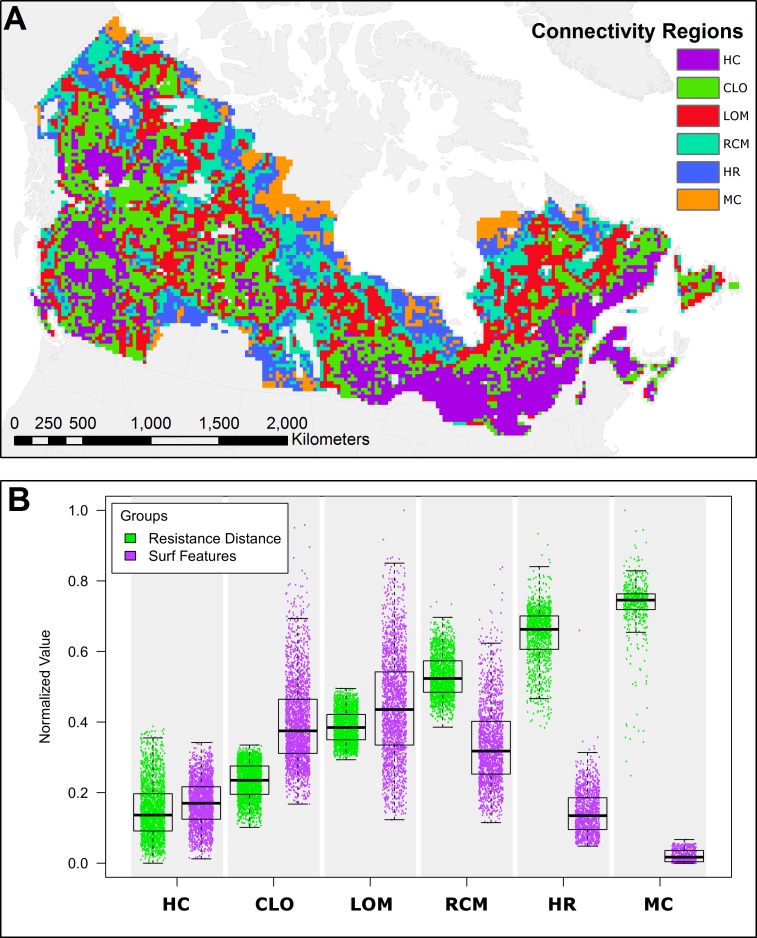
Forest connectivity regions of Canada and their characteristics. (a) Canadian forest connectivity regions; (b) resistance distance and structural complexity data for all tiles of each forest connectivity region. Abbreviations of forest connectivity regions: HC = Highly Connected; CLO = Connected with Large Obstacles; LOM = Limited Omnidirectional Movement; RCM = Restricted Corridor Movement; HR = High Resistance; MC = Minimal Connectivity.

**Fig 8:**
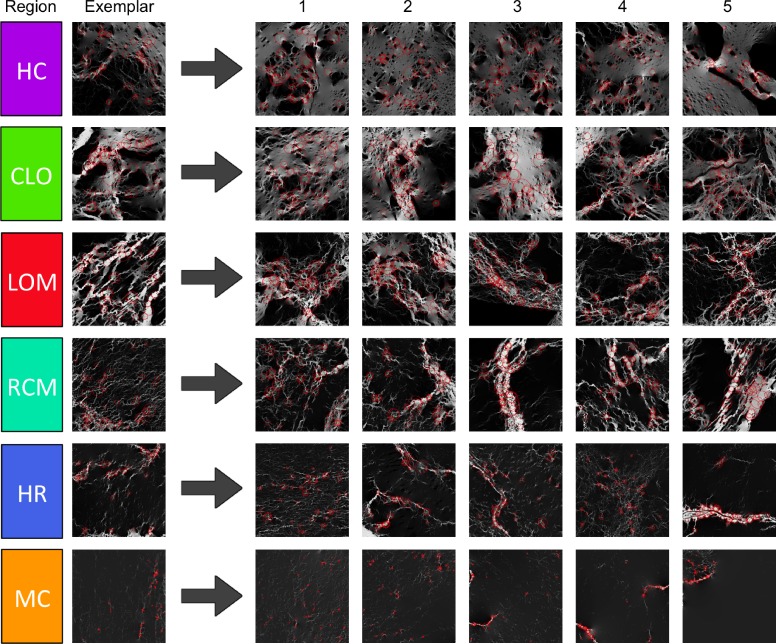
Exemplars of forest connectivity regions, with similar landscapes. The left column shows the Exemplar tile for each forest connectivity region of Canada. The right columns show the five tiles that are the most similar in resistance distance and complexity to each of the forest connectivity region Exemplars. Abbreviations of forest connectivity regions: HC = Highly Connected; CLO = Connected with Large Obstacles; LOM = Limited Omnidirectional Movement; RCM = Restricted Corridor Movement; HR = High Resistance; MC = Minimal Connectivity.

The spatial patterns of the resistance (Fig 4A) and complexity (Fig 4B) suggest the existence of forest connectivity regions at the national scale (Fig 7). Using the characteristics of resistance distance and structural complexity, landscapes of forested Canada can be clustered into groups (Fig 7) with Exemplars for each forest connectivity region (Fig 8). The first region (*HC*, *Highly Connected*) comprises a group of tiles with very low resistance distance values and medium complexity. Large areas of forest are visible and movement is possible in all directions with either wide corridors or multiple redundant corridors. The second region (*CLO*, *Connected with Large Obstacles*) generally surrounds region HC and has low resistance distance but a very large range of complexity, ranging from medium to high. It is composed of forested areas, but these areas have many large obstacles, which increases their complexity compared to region HC. Large areas of forest are visible, but they share the area with multiple large obstacles, such as lakes and marshes. Movement is possible in all directions, with multiple redundant corridors available. The third region (*LOM*, *Limited Omnidirectional Movement*) has intermediate values of resistance distance and also a large spread of medium to very high complexity. While the structural complexity of flow patterns in this region is similar to that of region CLO, its resistance distance is higher and its complexity indicates that obstacles and barriers to movement are more numerous. Forest connectivity in one of the cardinal directions may be restricted and sometimes requires large detours. The next region (*RCM*, *Restricted Corridor Movement*) has medium to high resistance distance and a large spread of medium to high complexity. The representative landscape for this class (Fig 8) is lower in complexity than the Exemplars in regions CLO and LOM because of its lower forest cover, possessing larger areas that have low current density and that are not detected as features by SURF. Tiles are either a maze of very small corridors or they are dominated by a few movement corridors that channel the majority of the current. Movement in certain directions is often impossible, as in tile RCM-3 in Fig 8. The next region (*HR*, *High Resistance*) has high resistance distance and medium complexity. This region has limited forest cover and omnidirectional movement. Sometimes a few corridors are visible on the tiles, but often, there’s only a patchwork of small corridors going around large obstacles. The last region (*MC*, *Minimal Connectivity*) has very high resistance distance and the lowest complexity. Movement inside forests across these tiles is costly, and current density aggregates in a few disconnected forest corridors. It is the region with the lowest forest content, with very few areas of high current density that can be detected as features by SURF.

## Discussion

Resistance distance is a fundamental output from circuit-based landscape analyses, yet it does not answer the important question about the locations of high and low flow within the landscape. Combining resistance distance and analysis of the current density map allows users to answer complex questions. By mapping resistance distance and using it spatially to interpret flow patterns, it is possible to answer questions such as “are these two areas with similar looking current density maps of similar resistance to movement?” The tiling approach allows for mapping resistance distance when using a tile size that is small enough to cover the study area with a large number of tiles in order to create a precise map.

### Visual interpretation of individual tiles

At the tile level, the results are directly tied to both the proportion and configuration of forest found on each tile. Tiles found in heavily forested areas (Fig 2A) show few major barriers to movement and a mostly homogeneous spread of current in forested areas. Tiles found in mountainous areas and areas with a lot of water bodies (Fig 2B and 2D) have major barriers that channel the flow of current through forested corridors. These tiles show high current density in available forested corridors and little or no current density in high resistance areas such as water bodies or rocky escarpments. For tiles that are sparsely forested with distinctly separated forest patches (Fig 5, Taiga Shield West Exemplar 1), there is no direct path that would allow current to flow freely through connected forests. Instead, current is forced to move among forest patches, concentrating there while spreading out spatially through high-resistance areas. Current density spreads evenly through the tile if no forest is present (upper right part of Fig 2C). While we modelled connectivity on these tiles as a part of this study, there is obviously no forest connectivity there in the real world as they are open areas typical of prairies, tundra and permafrost.

### Visual interpretation of multiple tiles and the omnidirectional current density mosaic

In this scenario, representing a forest dwelling organism, the resulting mosaic shows a map of possible forested movement paths over Canada. When viewed at multiple scales, the resulting mosaic (Fig 1) shows a map of possible connectivity pathways over Canada’s forested landmass. The mosaic clearly shows that the tiling method can scale to very large areas in a seamless and systematic fashion. The current density patterns are driven by aspects of composition and configuration of forest cover, with both affecting the features of landscape connectivity in a given area (Figs 1 and 2). In considering the connectivity mosaic, it is worthwhile to note that our objective was to generate landscape connectivity, which, as discussed in the Introduction, is not necessarily synonymous with habitat connectivity for target species of interest. Assessment of habitat connectivity for a particular species would likely require species-specific resistance weights tailored to represent the movement requirements of species or groups of species. This product therefore characterizes the connectivity of forested landscapes in Canada and as such, can be used to examine broad regional and national patterns of connectivity.

The classification of the resistance and complexity data into forest connectivity regions reveals countrywide patterns of forest connectivity. Within each ecozone, landscapes exhibited nearly the entire range of variability of both resistance distance and structural complexity (Fig 6). While useful for understanding landforms and land-cover proportions, ecozones were limited in their ability to precisely express the effects of the arrangement of these land covers on forest connectivity. Though forest amount and pattern vary widely among the thousands of landscapes, the resultant ease or difficulty of forest-based movements can be grouped into well-defined categories containing landscapes with similar characteristics, allowing for systematic assessments and comparisons across the very complex forested surface of Canada.

Although there is a well-defined pattern of connectivity when different areas are compared, for example on the north-south axis in eastern Canada, current density patterns must be interpreted carefully. First, current density patterns must be interpreted under the context of landscape connectivity and the simple resistance weights as parameterized. Moreover, fully forested areas have a homogeneous spread of current density around the landscape; few obstacles to movement are present and current is free to flow in any direction. Such homogeneous landscapes would have little spatial structure in the connectivity surface, and could look similar to landscapes with very little forest connectivity. Second, these are dynamic landscapes that change rapidly. In the 15+ years since the nominal date of the EOSD, many large areas that had recently been burned will now be vegetated. The EOSD data used here represents a snapshot in time, and any operational use of these results should both use updated land-cover data and explicitly consider connectivity as it changes through time.

### Tile-level analysis and quantitative interpretation of the connectivity mosaic

The resistance distance metric value returned by Circuitscape is useful to assess ease of movement on a tile. While this distance was first intended as a measure between two points or areas on the map, in this case it shows the ease of movement in the east-west direction and the north-south direction. The tile-level map of resistance distance allows for easy identification of connectivity types that might look similar when zoomed out on the current density map. Without the resistance distance map, these areas would need to be examined visually by exploring each area at high resolution in a GIS, but this approach would rely too strongly on human judgment and would be time consuming to execute. When using the resistance distance metric, is it easy to differentiate two such areas because the well-connected tiles will have a low resistance distance and the tiles with major obstacles to movement will have a very high resistance distance. While the map of tile resistance distance is very coarse, in areas as large as Canada it can convey a lot of information and help to quickly find areas of interest. If a large part of the analysis focuses on the comparison of values at the tile level, using smaller tiles with relatively large buffers would result in a map that functions somewhat like a moving window analysis in GIS software.

### Automatically detecting features of interest

SURF identifies a large set of features that correspond well with subjective judgements about the location of pinch points, barriers, and other features of the landscape, as described in McRae et al. [21]. The SURF algorithm detected abrupt changes in contrast and the different shapes of features, which is markedly different than other approaches that identify individual pixels of high current density. This process can be used to describe omnidirectional connectivity surfaces based on their complexity, helping users to spot areas with many features, such as pinch points, narrow corridors and major obstacles to movement. Tiles that have multiple large connectivity paths and low resistance distance tend to have a low number of features because most of the tile is made of a homogeneous spread of current density without a clear spatial structure to delineate. Tiles that are harder to traverse have a high number of visible pinch points, obstacles and narrow corridors, resulting in a high number of features. Tiles that have little to no forest, and thus little to no connectivity have the lowest number of features because they are characterized by homogeneous, low connectivity. There are clearly visible clusters of tiles with similar numbers of features all through Canada. Fig 6 shows that ecozones closer to the middle of the Canadian north-south axis have more features than other ecozones. As is visible in Fig 5, Exemplars from these areas have an almost even split between forest and non-forest areas and show the most complex connectivity patterns.

It is worth noting that SURF provides an orientation direction for each feature (shown as green lines in Fig 3D), which may be of interest in future work. Across hundreds of tiles and thousands of features, the direction perpendicular to the feature’s computed orientation consistently corresponded with our own judgement of the implied movement direction in that part of the landscape. Though we have not pursued that here in detail, it is worth considering that SURF might be used to reveal the varying directions of overall movement within a landscape.

### New Metrics in omnidirectional connectivity surfaces

Neither resistance distance nor the structural complexity information are alone sufficient to characterize forest connectivity in these omnidirectional surfaces (Fig 7). For example, landscapes in the Highly Connected and Connected with Large Obstacles categories have similar, low values of resistance distance, indicating that landscapes of both connectivity types can be crossed easily. The structural complexity value, however, which responds more directly to the configuration of land cover on the tile, shows substantially more pinch points and corridors in landscapes with these obstacles. More broadly, the results suggest that landscape functionality and landscape structure are not necessarily linearly related, consistent with other recent attempts to quantify connectivity in current density surfaces [48]. Moreover, both the simple estimate of structural complexity and the SURF-identified features themselves could serve as new and needed [49] quantifications of landscape pattern.

### Limitations and future work

This study is limited in several important ways. First, the EOSD land-cover map is a considerable simplification of reality, and the binary forest/nonforest map is an even further simplification. Given the very complex response of Circuitscape to specific patterns of configuration as well as composition in land-cover maps (e.g., S1 Fig and [31]), we saw this simplification as an important first step in understanding and organizing the many landscape patterns that emerged across forested Canada. Second, land cover mapped in the EOSD represented only a snapshot in time; fire, human action, and regrowth have changed the forest cover since its nominal date of 2000.

In the light of these limitations, future work should focus on the stability of a given landscape’s omnidirectional connectivity map and the derived connectivity measures in more subtly mapped and changing environments. With the ever-increasing availability of computing power, it should soon be possible to do these computationally very demanding analyses for longer land-cover time series, in which changes in connectivity and landscape complexity could be assessed year after year from land cover generated from cloud-free, satellite image composites [50, 51]. For example, the method could be applied to areas with severe disturbances (e.g., fires), to quantify variations of connectivity patterns over time. These effects could be compared to those of smaller and more frequent disturbances, to explore how these phenomena could influence the overall connectivity in large areas.

Significant differences in connectivity through time among landscapes with these histories could inform decisions about future management. Future work can employ the measures described here to study under what specific conditions the resistance distance and detected features of omnidirectional connectivity surfaces vary significantly. That next step would allow users to understand the implications of parameterizing a resistance surface more subtly—for example, when estimating levels of permeability for multiple organism types [24, 48], when combining surfaces for species with different habitat requirements [25], or when pursuing the many other choices that can drive land-surface parameterization [52, 53]. Finally, tile-level mapping of resistance distance and connectivity features will allow for comparison with established land-cover metrics, such as those supported by Fragstats [54] to discover new relationships with connectivity. The new possibilities offered by these connectivity metrics can help guide future modeling efforts and scenario development.

## Conclusion

Through tiling of omnidirectional connectivity maps across Canada and the development of automatic feature detection, this manuscript demonstrated a tractable way to identify some of the key characteristics of interest in Circuitscape-based maps across large areas. While resistance distance provides an informative composition-driven descriptor of a given landscape, the feature detection described in this manuscript presents a new spatially explicit configuration-based assessment of a landscape’s current flow at multiple scales. This combination of metrics of composition and configuration, if further refined and explored in future work, might contribute to a foundation of standard measures on circuit-based flow maps, analogous to how FRAGSTATS provides pattern information, with caveats [49, 55, 56], about land-cover maps. With these measures, connectivity mosaics allow for the better identification of important regional and local connectivity patterns, informing science and guiding researchers and managers in support of land management processes.

With the help of image recognition software, we can now assess the complexity of broad connectivity patterns to detect areas with multiple barriers and obstacles, as well as areas of low structural complexity. These fine-resolution characterizations over a large area allow for detailed local studies as well as broader regional/national understanding of the links between connectivity and a range of ecological processes. Here, we have shown some initial opportunities and insights, but certainly see additional and myriad possibilities in the application of these quantitative connectivity surfaces in the future.

## Supporting Information

S1 Fig Three landscapes that illustrate the complex relationship between landscape configuration and the characteristics of the resulting omnidirectional connectivity surface. Here, three landscapes are highlighted whose composition is half forest and thus, the same total resistance distributed unevenly around the tile. These landscapes were selected from the 121 Canadian landscapes having between 49.5% and 50.5% forest cover in the NLCD. Despite having near-identical landscape compositions, the configuration of the landscapes strongly affects the ease of passage in each. Among the 121 half-forested landscapes, the images are of (top panel) the 20^th^ percentile of resistance distance (that is, easier to cross); (middle panel) the 50^th^ percentile of resistance distance (that is, moderate crossing difficulty); (bottom panel) the 80^th^ percentile of resistance distance (that is, harder to cross). These landscapes were also distinct from each other in the broader context of all 9923 tiles in Canada: the landscape in the top panel was in the 37th percentile of resistance distance; the middle landscape was at the 49^th^ percentile, and the bottom tile was in the 71^st^ percentile of the full set of Canadian landscapes.
(PNG)
