# Supplementary figures and images for: Forest Connectivity Regions of Canada Using Circuit Theory and Image Analysis
Source: PLoS One. 2017 Feb 1;12(2):e0169428. doi: 10.1371/journal.pone.0169428 (PMC5287482; doi:10.1371/journal.pone.0169428)

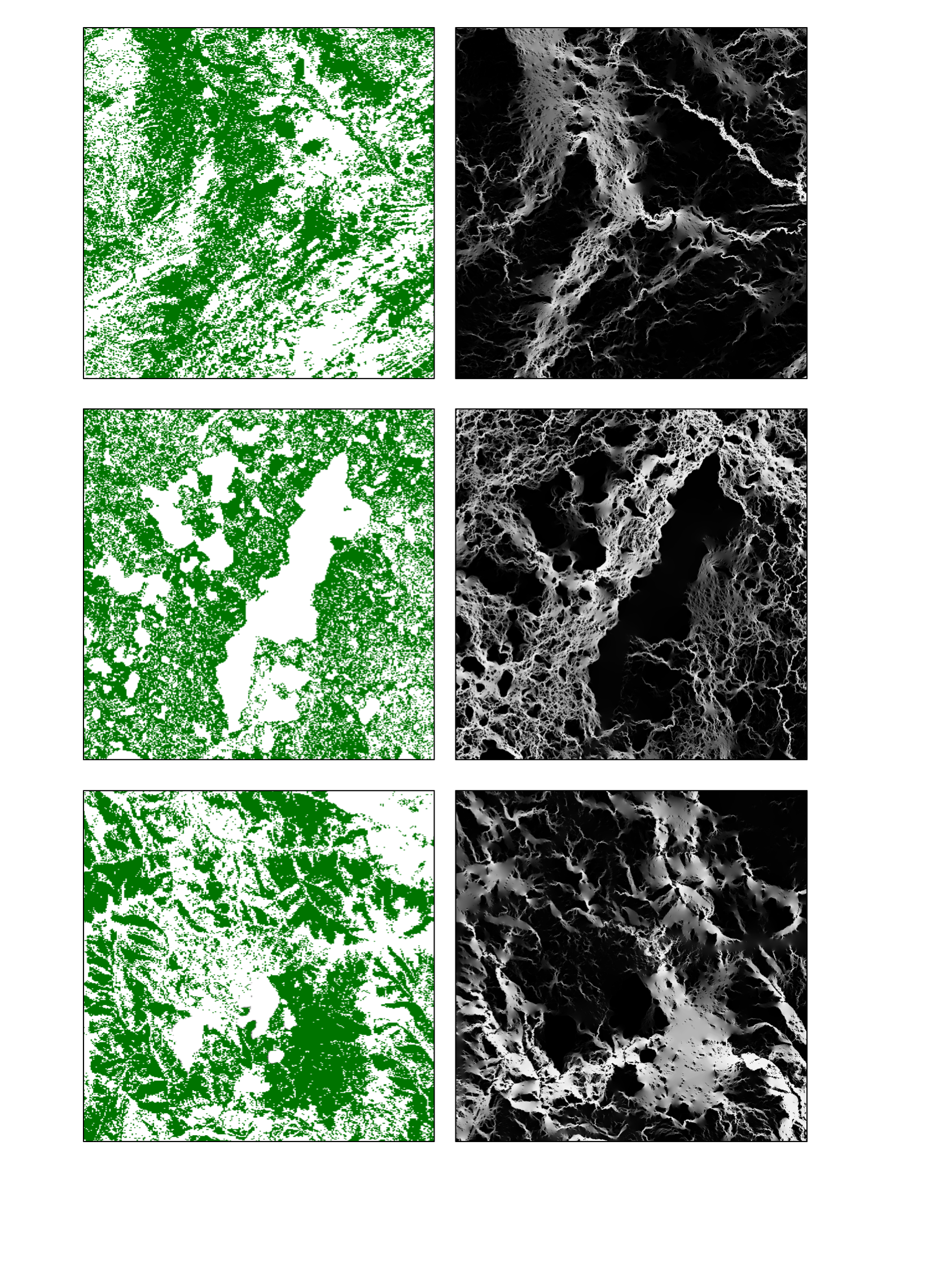

Supplement: S1 Fig — Here, three landscapes are highlighted whose composition is half forest and thus, the same total resistance distributed unevenly around the tile. These landscapes were selected from the 121 Canadian landscapes having between 49.5% and 50.5% forest cover in the NLCD. Despite having near-identical landscape compositions, the configuration of the landscapes strongly affects the ease of passage in each. Among the 121 half-forested landscapes, the images are of (top panel) the 20th percentile of resistance distance (that is, easier to cross); (middle panel) the 50th percentile of resistance distance (that is, moderate crossing difficulty); (bottom panel) the 80th percentile of resistance distance (that is, harder to cross). These landscapes were also distinct from each other in the broader context of all 9923 tiles in Canada: the landscape in the top panel was in the 37th percentile of resistance distance; the middle landscape was at the 49th percentile, and the bottom tile was in the 71st percentile of the full set of Canadian landscapes. (PNG) [file pone.0169428.s001.PNG]
